# English as a Foreign Language Learners’ Well-Being and Their Academic Engagement: The Mediating Role of English as a Foreign Language Learners’ Self-Efficacy

**DOI:** 10.3389/fpsyg.2022.882886

**Published:** 2022-05-23

**Authors:** Hui Jia

**Affiliations:** Henan Institute of Science and Technology, Xinxiang, China

**Keywords:** academic engagement, EFL learners’ self-efficacy, well-being, positive psychology, EFL

## Abstract

Well-being is a crucial necessity within the educational setting that is also taken into account as a central aspect of people’s inclination in the subject of positive psychology (PP) study which is vital for the learners’ affective equilibrium and proper development and improvement. Likewise, learners’ engagement has been demonstrated to have a fundamental function in education. A great amount of attention has been given to this concept and its possible indicators because of its role at the core of learners’ educational achievement. Alternatively, it is commonly maintained that self-efficacy has turned into a significant mental concept enhancing the educational cycle and educational presentation that influences learners’ decisions regarding their educational assignment and manners and their way of thinking and feeling when it comes to education. This review attempts to survey the role of learners’ self-efficacy as a mediator on their well-being and academic engagement. In conclusion, some suggestions and commendations have been proposed for language-teaching participants in scholastic situations.

## Introduction

A large number of learners got their individual and environmental reasons for getting dropped out of school, and dropping out means lack of engagement and is a quickly increasing incident these days (Lippman and Rivers, 2008); however, the main cause is poor achievement due to reduced academic motivation, referring to the process, where targeted activities are energetic, directive, and sustainable (Schunk et al., 2010). Many inquiries are carried out to ascertain the aspects related to poor or good educational presentation, among which well-being has been considered as a clearly significant one (Mercer, 2020). Indeed, well-being is one of the key notions inspected in the domain of PP since it is related to a broad scope of educational and mental results involving low stress, boredom, and exhaustion in addition to greater degrees of success, constructive behavioral manner, and the capability of adapting to alterations (Derakhshan et al., 2021).

Associated with life fulfillment, well-being includes self-worth, constructive connections with others, independence and capability, and objective-orientedness; furthermore, an emphasis on individual development is crucial to learners since it leads to maximum functioning and engagement (Steele and Fullagar, 2009). Well-being which is at the heart of PP can be scrutinized from the perspective of personal complexities (Wang et al., 2021) and the learners’ well-being has been informed as the key construct of their educational accomplishment and those learners with higher well-being usually got better marks and are unlikely to encounter failure (Yang, 2021). Indeed, pleasure or well-being is a result of learners’ engagement and ideal presentation in existential hardships of life as motivational and behavioral dimensions useful for studying and adapting a person to an educational environment is called engagement (Salmela-Aro et al., 2016).

Similarly, academic engagement as one of the constructive psychological concepts is predominantly vital in higher education due to its constructive relations with different learner consequences, their mental health, accomplishment, and enthusiasm (Rogers et al., 2017). The significance of learners’ engagement in language learning studies was identified by educators. It’s evident that large numbers of learners get fatigued, and lack motivation and engagement, i.e., indifferent to academic and social dimensions of school life (Appleton et al., 2008). Learners’ engagement is a roughly new, extensive, and multidimensional structure, that is changeable and refers to the dedication to teachers and school-associated works (Reschly et al., 2014). Learner engagement is conceptualized as individuals’ willingness to involve in educational activities in terms of behavior, emotion, and cognition (Sharkey et al., 2008).

Thanks to the reality that learners’ engagement has a crucial role in growing their studying results (Carver et al., 2010), encouraging the learners to get engaged within the studying procedure has constantly been a concern for educators in all academic fields and it is determined to be vital for studying, educational performance, endurance, retention, and educational fulfillment (Gunuc and Kuzu, 2015). It is worth mentioning that one can measure learner achievement through the degree of actively engaging and taking part in educational activities in the class and self-developing activities through after school activities and interaction in diverse activities necessitating learners’ self-efficacy as a vital element in helping the engagement of learners (Muallifah et al., 2020).

Undeniably, learning a language includes different emotional factors, a sense of self-efficacy is one of them that has turned into a significant issue in teaching, and it means individuals’ certainty of their own talent to perform a specific assignment (Genç et al., 2016). As scholars believe, self-efficacy is a type of learners’ confidence in his/her capability to be optimistic in managing all the challenges felt in school so that they can broaden tactics to comply with the process of learning nicely (Linnenbrink and Pintrich, 2003) and it is an internal resource associated with learners’ principles in performing that is inferred to a learners’ assurance in their aptitudes and capabilities to accomplish the anticipated objectives through the activities presented (Chang and Chien, 2015). Self-efficacy refers to a widespread mental necessity that manages a person’s cognition, affections, and selections about mental well-being (Komarraju and Nadler, 2013). In addition, self-efficacy can also affect how learners feel regarding themselves, and if they correctly complete their targets throughout life (Bartimote-Aufflick et al., 2016).

Self-efficacy influences the cognitive and bodily effort individuals put into activities, persistence duration when faced with challenges, and learning and achievement levels. High-efficacy learners are willing to determine difficult challenges, work hard, persevere when encountering a problem, and restore effectiveness after difficulties (Schunk and Mullen, 2012). Efficacy refers to a strong index of academic efficiency, educational fulfillment, and a large number of different major elements (Fathi et al., 2020) characterized as people’s beliefs considering their capability to exhibit particular levels of performance with an influence on occasions affecting their lives (Bandura, 2010). Of course, self-efficacy affects learner incentive as high self-efficacy enhances learner engagement and achievement (Van Dinther et al., 2011) and it pertains to the sensation of self-assurance in his ability and is strongly diagnosed by the desire to study more or the desire to take part in challenging tasks in comparison to overall interest tasks (Han and Wang, 2021).

Moreover, scholars in the field of social cognition studied the functioning and consequences of the cognitive and emotional processes that are assumed to cause engagement (Schunk and Pajares, 2005). Indeed, a large number of language teaching beneficiaries hold that a crucial element in enhancing second language learners’ final achievement is to foster participation in them (Mercer, 2019). Academic studies indicate that learners who feel academically effective are skillful and interested in learning, determine learning targets, employ efficient learning tactics, supervise understanding, assess progress toward goals, and build an encouraging setting (Schunk and Pajares, 2005). Given the common issue of disengagement among Chinese English as a Foreign Language (EFL) learners, there has been increasing interest in bringing PP into this domain (MacIntyre et al., 2016). While the prominence and status of constructive variables such as perseverance, efficacy, well-being, commitment, and resilience are certified in some prior investigations that predict necessary educational results (Elahi Shirvan et al., 2021; Sulis and Philp, 2021), there is still a dearth of research for presenting how ones can be authorized by paying attention to these issues. Among them, self-efficacy has been deemed to act as both facilitator and interpreter of constructive education results that are proved in numerous experiential works (Alivernini and Lucidi, 2011; Wilson and Narayan, 2016) and even though self-efficacy is considered to be a vital issue in educational accomplishment and its direct impacts on different constructs are well-acknowledged, to the best of the researcher’s knowledge, its role has not been extensively investigated on learners’ engagement and well-being in the language learning context that is taken into account in this study.

## Review of Literature

### Student Engagement

At first, engagement was considered as a framework employed merely in job environments. Step by step, teaching scholars have discovered that this also applies to academic environments. They described learner engagement as a mixture of pleasure, interest, and focus on the process of learning. In PP, engagement alludes to being greatly interested, immersed, or attentive in everyday tasks. It is the condition in which a person is completely fascinated and immersed in activities and utilizes their curiosity and abilities to the analysis and a great degree of engagement in an activity alludes to a flow or the general sense of battling it out (Seligman, 2018). This happens in the class or any time learners are busy with their academic assignments such as studying. Learner’s engagement has a significant role in educational psychology when it comes to academic achievement and intrinsic incentives (Salanova et al., 2010).

Based on the findings, learners with academic engagement have higher educational progress considering better GPAs and higher lesson scores in comparison to learners with no engagement (Ketonen et al., 2016). In addition, it was found that learners with engagement have been more positive about their career preference, while learners with no engagement had no interest or were uncertain regarding their professional route. Several scholars have conceptualized learner engagement as a concept having many aspects with three different dimensions, namely, cognitive, behavioral, and emotional dimensions (Grier-Reed et al., 2012; Gunuc and Kuzu, 2015). Engagement extensively helps to the fulfillment of learners’ educational success and it allows learning to happen and is a predictor of their educational performance and normal development (Reeve, 2012). Learners with engagement have inherent motivation to study, frequently attend courses, and engage in educational activities and they are curious, inclined to embody learning difficulties, and feature extra power to learn (Salanova et al., 2010).

School engagement in the literature refers to a meta-construct constituted of dimensions in terms of behavior, cognition, and affection (Wang et al., 2021). Behavioral engagement involves actively participating and engaging the learner in social forums, class interplay, and learning, in school as well as at home, and after-school activities associated with school (Hiver et al., 2021). Cognitive engagement, however, means learners’ individual depletion in studying activities, namely, self-regulation, the dedication to become proficient in studying, and using learning tactics (Sedaghat et al., 2011). Affective engagement involves the emotional elements of engagement, namely, pleasure, backing, attachment, and approach toward educators, friends, studying, and also schools (Reeve, 2012). The elements of school engagement in terms of affection, cognition, and behavior are recommended to seize the pertaining but separate developmental aspects that help a learner’s lively engagement during the school time (Wang and Eccles, 2012). As stated by Appleton et al. (2008), engaged learners constructively discern the educational cycle and endeavor to attain class content. The definition provided for educational engagement is a positive mood containing energy, commitment, and attraction to studying manifested by aspects of power and identification (Siu et al., 2014). Research on engagement mostly concentrates on analyzing variations in inter-subject, thinking about this variable as an enduring trait (Bakker et al., 2015).

### Well-Being

The fundamental aim of PP is to enable pleasure and well-being and PP, with its attentiveness to well-being, does not disregard people’s problems, but it faces them from the outlook of social power instead of weakness (Seligman, 2018). Associated with life fulfillment, well-being includes self-worth, constructive connections with others, independence and capability, and objective-orientedness; furthermore, an emphasis on individual development is crucial to learners since it leads to maximum functioning and engagement. Well-being consists of two critical viewpoints, namely, hedonia and eudaimonia. Getting close to environmental and affective relief, having a pleasing influence, and lack of unpleasing influence is called hedonia, which means general satisfaction. Alternatively, trying to self-grow constantly with values and complete mental actualization is called eudaimonia (Giuntoli et al., 2021). Both of the key terms emphasize an optimistic feeling; but the hedonic approach concentrates on people’s affections, pleasure, and happiness at a certain time, whereas the eudemonic approach is (Disabato et al., 2016).

The primary objective of PP is to ease pleasure and subjective well-being (Seligman, 2018). Optimistic psychology experts try measuring well-being from an optimistic perspective. The optimistic mind study motion defines well-being as “optimistic and maintainable traits” which allow people and corporations to make efforts and grow (Greenier et al., 2021). Seligman (2018) enlightened that well-being has several components and multiple measurable elements exist that help develop the well-being conceptual context known as the theory of PERMA with five themes, namely, positive affections, relations, engaging, meaningfulness, and attainment. Positive emotions include satisfaction, positivity, and well-being, which are considered as part of the hedonic spectrum of emotive conditions that work as pointers of success since they can aid individuals with prospering and can be instructed and enhanced (Fredrickson, 2001).

Engagement is commonly referred to as a type of flow or profound participation that is essentially intended to be inspiring throughout the accomplishment of an assignment (Derakhshan, 2021). Goal setting, monitoring, and achievement increase well-being during an entire life course (Heckhausen et al., 2010). Constructive connections imply a feeling of being societally embraced, recognized, and empowered, and enjoying one’s societal connection. Societal support is linked to constructive results of mental and physical well-being, in addition to overall well-being (Greenier et al., 2021). Meaning is the notion that an individual’s life has perseverance and a path in the course of life. It involves being connected to something bigger as well as positive emotions in various age ranges (Yang, 2021). Achievement is generally connected to goal setting, development, and having the skill to succeed, thereby endeavoring for well-being (Fredrickson, 2001).

### Self-Efficacy

Self-efficacy can be assumed as a component of self-notion, which alludes to an individual’s recognition of his or her ability to effectively present a specific assignment. In a broader sense, educational self-efficacy is the recognition of a learner’s capability of achieving the intended degree of educational presentation (Huang, 2011). The skill to conform to new occasions and needs increases when people have a greater feeling of self-efficacy (Seifalain and Derakhshan, 2018). Self-efficacy refers to a firm element in determining how a person would do, contemplate, and react when faced with challenging occasions. It is essential in growing learners’ characteristics to promote their learning procedure (Thompson and Verdino, 2019). The self-efficacy concept comes from Bandura’s authentic work within the social studying assumption, and inside that assumption, Bandura described studying as intellectually gaining information *via* processing data gained by observation of others (Miller, 2011). Indeed, the assumption of social learning determines three pertaining learning elements, such as psychological characteristics, behavior, and setting. Bandura commenced noting that people’s sense of success and ability to continue despite tasks assumed a critical section in studying *via* working with the social learning assumption. Through such an issue, Bandura raised the self-efficacy assumption (Miller, 2011).

Self-efficacy is a central issue in describing how people act, contemplate, and respond in case of difficult instances (Downes et al., 2017). Studies such as Salanova et al. (2010) indicated that convictions on efficacy affect presentation and involvement in exercises, and ultimately, educational engagement. Moreover, it is worth mentioning that self-efficacy aids in presuming and making endeavors that are needed to carry out different types of assignments effectively (Whannell et al., 2012). Learners’ degree of self-efficacy inside the learning trend can be an optimistic experience for their educational difficulties which reinforces their belief in gaining and achieving a specific topic to determine an educational specialty (Los, 2014).

Self-efficacy is a major factor in the theory of social cognition because individuals need to understand themselves and their abilities to manage their behaviors (Pajares, 2009). Consistent with him, self-efficacy ideas offer a firm basis for improving people’s encouragements, well-being, individual achievement, and taking risks, besides reducing anxiety levels. Self-efficacy falls into three primary kinds: self-regulatory, i.e., self-efficacy consisting of the potential to withstand peer strain and refrain from works involving excessive hazard, social, i.e., self-efficacy consisting of the potential to shape and preserve relationships, to be self-confident and engaged in leisure time works, and educational, i.e., self-efficacy consisting of the potential to effectively pursue education, control the activities of learning, and satisfying one’s own expectancies (Branscombe and Baron, 2016).

## Conclusion

Centered on the PP theory, learners are interested in looking for well-being and engagement in the learning procedure and because of the noteworthy contribution of these concepts in their language success, it has been at the center of many researchers’ attention (Reeve, 2012) and it is argued that a greater degree of learners’ engagement in a language class is significant since it is capable of predicting learners’ advancement, facilitates education, and it has a significant function in building learners’ critical thinking abilities, problem-solving abilities, and other intellectual skills, and improves their conscientiousness in different domains (Tytler et al., 2008). Moreover, educational self-efficacy is not directly associated with the presentation, but successfully predicts a higher degree of educational engagement. Although self-efficacy is likely to be linked to a greater belief in achievement, it will not certainly lead to enhancement in the presentation if educational engagement is absent.

Furthermore, the literature reviews proposed that the learners’ self-efficacy is greatly connected to every dimension of learners’ engagement, due to the fact that learners’ self-efficacy affects their degree of inspiration, their perseverance during hardships, and their selection of assignments. Indeed, higher self-efficacious learners are consequently inclined to have greater degrees of engagement in diverse tasks and actions. Alternatively, those learners who have low self-efficacy avoid certain tasks or pretend their ineffectiveness in completing their goals that result in a lack of engagement. So, the role of self-efficacy is dominant because learners with a great degree of self-efficacy in education and in carrying out a designated assignment effectively engage in the assignment as opposed to evading it, persevere in times of hardships, and attempt various techniques, all of which result in more engagement that also bring about success.

Moreover, self-efficacy has a constructive relationship with engagement because it increases the intention to spend more vigor and endeavor to accomplish an assignment or a duty; therefore, it increases participation and immersion in the assignment (Ouweneel et al., 2011). Efficacy impacts learners’ motivation and a great level of efficacy enhances their engagement and achievement that is related to the sensation of self-confidence in people’s capability that can be strongly diagnosed with the desire for further study or the desire to take part in tasks perceived as challenging in comparison to tasks desired (Vecchio et al., 2007). Efficacious learners are more prone to adjust their inspiration by determining objectives for themselves and are more prone to be engaged so determining objectives and arranging could increase engagement through the achievement of objectives (Diseth, 2011). Undeniably, as there was demonstrated in intervention research among learners, controlled alterations in degrees of self-efficacy are connected to parallel alterations in degrees of vigor and commitment (Bresó et al., 2011).

Besides, the review indicated that high self-efficacious learners’ experience constructive well-being in their learning process, and as a result, they are more encouraged and more efficacious in their education (Seligman et al., 2009). Undoubtedly, since learners’ well-being is crucial to their engagement, they must be encouraged with constructive elements to improve their educational presentation and individual development (Durón-Ramos et al., 2018). Feelings are regarded as the trigger for managing behavior, thereby suggesting that feelings are an impetus for educational engagement. When learners encounter constructive affection such as pleasure and interest in school activities, they are prone to display more vigor and endeavor, which affects their demeanors toward education and motivates them to engage more in educational assignments (Fredricks, 2011). It is also asserted that well-being could be a potential indicator of the advancement of learners’ educational engagement since constructive feelings can aid them in visualizing objectives and activating their endeavors to achieve them. A constructive connection between overall self-efficacy and mental well-being confirms outcomes that a strong sense of individual efficacy significantly increases the standard of mental functioning, particularly, a sense of life self-actualization and psychological health.

## Implications and Future Directions

The results of this research offer several perceptions of major tactics that educators and school heads can follow to improve participation also, intervening programs aiming at improving school participation among learners must include tactics to ease self-efficacy and well-being and lessen failure phobia. Consequently, educators and school administrators, advisors and psychologists, and society need. Given the reviews studied, the conception of educational self-efficacy has a primary function in predicting all dimensions that build up educational achievement. Furthermore, educators should recognize this fact and offer learners assignments that pose a specific difficulty and simultaneously increase their sense of ability and inspire them to be involved in the class tasks. Educators need to attempt to inspire learners to plot their studying tasks, by supervising them and managing the setting to guide their studying activities. Therefore, people’s low self-efficacy may impede their learning procedure despite having the brilliant ability. Improving learners’ self-efficacy *via* active techniques of learning is deemed to play a major role in the process of learning.

Through executing active studying, learners can affect and take part vigorously in learning activities in such a way that instructing goals can have better achievement. Learning actively will enable learners to find the satisfactory opportunity in solving any problems pertaining to the course content, to structure themselves to satisfy their needs as learners, and have the courage to encounter challenges properly. Moreover, learners dare to speak publicly with no fear and have faith in conducting their tasks and continually evaluate themselves for the better through learning actively (Jeong et al., 2019).

Just as participation is strengthened through difficult sources and requirements and has optimistic results for learners’ great fulfillment (Salanova et al., 2010), schools are recommended to proceed with educational plans that consistently increase learners’ well-being while at the same time attaining related experiences and abilities. Educators need to inspire high levels of learner efficacy since those with high degrees of self-efficacy make hard efforts to study and succeed. Also, teachers must organize a vigorous and enthusiastic circumstance for the learners in the classroom to preserve their self-efficacy that can consequently improve their engagement and well-being. Consequently, by evolving students’ self-efficacy, faculty members can certify the learners’ greatest levels of well-being engagement. Directors of higher education must pay attention to learners’ improvement of self-efficacy because learners’ self-efficacy has a predictor and intervening function regarding their success, incentive, and participation that results in language learning achievement. Consequently, the relevant informative study groups and seminars have to enhance learners’ understanding and expertise regarding self-efficacy, and the pertaining elements. In this regard, numerous projects and tactics exist for developing, fostering, and instructing competencies.

In this review, student engagement and well-being are inspected by internal elements, such as self-efficacy. However, more studies can be done in the future to consider external factors, and for information retrieval, subsequent studies can be conducted *via* triangulation and engaging other schools at all levels of proficiency. The scholar can then relate different variables associated with learner participation, such as individual differences, self-regulation, and engaging parents. Moreover, this review paves the path for more studies on self-efficacy and well-being and their relevant elements that can help in making novel discoveries that are generalized to all language learners.

## Ethics Statement

The studies involving human participants were reviewed and approved by the Henan Institute of Science and Technology Academic Ethics Committee. The patients/participants provided their written informed consent to participate in this study.

## Author Contributions

The author confirms being the sole contributor of this work and has approved it for publication.

## Conflict of Interest

The author declares that the research was conducted in the absence of any commercial or financial relationships that could be construed as a potential conflict of interest.

## Publisher’s Note

All claims expressed in this article are solely those of the authors and do not necessarily represent those of their affiliated organizations, or those of the publisher, the editors and the reviewers. Any product that may be evaluated in this article, or claim that may be made by its manufacturer, is not guaranteed or endorsed by the publisher.
